# Target therapy for high-risk neuroblastoma treatment: integration of regulatory and scientific tools is needed

**DOI:** 10.3389/fmed.2023.1113460

**Published:** 2023-07-14

**Authors:** Adriana Ceci, Rosa Conte, Antonella Didio, Annalisa Landi, Lucia Ruggieri, Viviana Giannuzzi, Fedele Bonifazi

**Affiliations:** Research Department, Fondazione per la Ricerca Farmacologica Gianni Benzi Onlus, Bari, Italy

**Keywords:** neuroblastoma, rare diseases, drug development, target therapy, pediatric regulation

## Abstract

**Introduction:**

Several new active substances (ASs) targeting neuroblastoma (NBL) are under study. We aim to describe the developmental and regulatory status of a sample of ASs targeting NBL to underline the existing regulatory gaps in product development and to discuss possible improvements.

**Methods:**

The developmental and regulatory statuses of the identified ASs targeting NBL were investigated by searching for preclinical studies, clinical trials (CTs), marketing authorizations, pediatric investigation plans (PIPs), waivers, orphan designations, and other regulatory procedures.

**Results:**

A total of 188 ASs were identified. Of these, 55 were considered ‘not under development' without preclinical or clinical studies. Preclinical studies were found for 115 ASs, of which 54 were associated with a medicinal product. A total of 283 CTs (as monotherapy or in combination) were identified for 70 ASs. Of these, 52% were at phases 1, 1/2, and 2 aimed at PK/PD/dosing activity. The remaining ones also included efficacy. Phase 3 studies were limited. Studies were completed for 14 ASs and suspended for 11. The highest rate of ASs involved in CTs was observed in the RAS-MAPK-MEK and VEGF groups. A total of 37 ASs were granted with a PIP, of which 14 involved NBL, 41 ASs with a waiver, and 18 ASs with both PIPs and waivers, with the PIP covering pediatric indications different from the adult ones. In almost all the PIPs, preclinical studies were required, together with early-phase CTs often including efficacy evaluation. Two PIPs were terminated because of negative study results, and eight PIPs are in progress. Variations in the SmPC were made for larotrectinib sulfate/Vitrakvi^®^ and entrectinib/Rozlytrek^®^ with the inclusion of a new indication. For both, the related PIPs are still ongoing. The orphan designation has been largely adopted, while PRIME designation has been less implemented.

**Discussion:**

Several ASs entered early phase CTs but less than one out of four were included in a regulatory process, and only two were granted a pediatric indication extension. Our results confirm that it is necessary to identify a more efficient, less costly, and time-consuming “pediatric developmental model” integrating predictive preclinical study and innovative clinical study designs. Furthermore, stricter integration between scientific and regulatory efforts should be promoted.

## Introduction

Neuroblastoma (NBL) is the commonest pediatric extracranial solid tumor (1). While local low-risk NBL can be controlled with a high rate of cure (2), there is an urgent need to develop new treatment options for the high-risk NBL that still represents a leading cause of death from cancer in children (3) due to chemoresistance and frequent metastatic relapse. Optimized regimens incorporating emerging new molecules targeting NBL and other pediatric cancers are under development (4). By the end of 2020, the number of pediatric oncology medicinal products (MPs) increased; however, this increase was lower than what was observed for adult products. In fact, a total of 174 oncology medicinal products have been approved by the European Medicine Agency (EMA) in the period 2007–2020, but only 35 (20%) have been approved for use in children in the same period (5).

The scarcity of pediatric medicinal products is not surprising. In fact, the experience accumulated in the recent years demonstrates that the process of approval for a “pediatric” or a “rare diseases” product is particularly long and complex and affected by several research barriers and gaps (5, 6). Moreover, commercial and financial issues represent possible obstacles for the complete and timely development of such products (7).

The lack of novel pediatric oncology MPs appears in huge contrast with the numerous ASs identified in recent years targeting tumor-specific genomic abnormalities with the greatest potential to be developed as effective cancer therapies (8, 9). In some cases, genomic abnormalities identified in pediatric cancer are different from those correlated to adult cancers; in other cases, they are similar. However, while several new cancer agents are emerging worldwide, only rarely, pediatric indications were included in the adult drug or in pediatric-specific development programs (i.e., dinutuximab, representing the only product approved for NBL).

Focusing on NBL, the number of novel ASs targeting genetic mutations and molecular pathway aberrations, such as MYCN, BIRC5, PHOX2B, as well as epigenetic factors, tyrosine receptor kinase (TRK) inhibitors, and other targets, is impressive, as summarized in relevant studies (10–16). However, the number of ASs that has reached the patients (i.e., included in frontline therapies or granted with a marketing authorization—MA) is very low. Indeed, the extensively very high number of potentially druggable targets has been considered a relevant barrier to successfully developing new MPs (17). The project ACCELERATE, faced with these issues, started a prioritization initiative to identify more promising ASs to be advanced in the developmental process (18). Clarification on preclinical aspects, such as the availability of reliable “pediatric cancer model” and “proof of concept” for pediatric indication, emerges as a relevant premise, and a proposal to define a “preclinical package”, shared with regulators, is under discussion (19). In line with these initiatives, the Neuroblastoma New Drug Development Strategy (NDDS) activated a prioritization process within ASs targeting NBL that ended in 2020 (20) with 40 genetic targets evaluated and 23 ASs prioritized for further development.

To bring a pediatric MP onto the market, both in the European Union (EU)^1^ and the United States of America (USA) (21), there is the obligation for the sponsors of a new AS or of a still in-patent MP to complete a detailed plan of pediatric studies [i.e., the Pediatric Investigation Plan, PIP in the EU (22), and the Pediatric Study Plans—PsPs in the USA (23)], as agreed with the concerned regulatory agencies. This obligation may only be waived for one of the legally accepted reasons (24), such as (1) a product intended for a disease or condition not existing in a specified age subset; (2) an expected lack of safety or efficacy; or (3) a lack of significant therapeutic benefit in the pediatric population (23).

Among these, the waivers agreed to on the grounds that the proposed drug is intended for the treatment of adult cancers, not to be used in children, have been largely granted in the EU, but in July 2015, the Pediatric Committee (PDCO) adopted a review of the class waiver list (25) aimed at limiting these types of waiver applications.

In the USA, the RACE for Children Act (FDA Reauthorization Act) (26) provides that pediatric studies must be conducted if the mechanism of action (MoA) of the AS may be relevant for a pediatric cancer indication independent of the adult indication. Currently, the transition to a similar MoA-based regulatory approach is being favorably considered within the European Commission (EC) proposal for revision of the Pediatric and Orphan Regulation (6).

In addition, a number of regulatory tools, procedures, and incentives [i.e., the orphan designation (27), Scientific Advice/Protocols Assistance, research funds, conditional approval, and PRIME designation] were included in the EU legislative framework to support and accelerate the development of MP particularly relevant for patients, especially in case of innovative products. All these tools are fully applicable to NBL.

Similar to the USA, the EU legislation^1^ was introduced with the aim to provide children with medicines appropriately assessed for pediatric use on the basis of a full developmental plan that includes preclinical and clinical investigations. Compliance with the current legislation may be challenging in the pediatric oncology field since some regulatory processes and procedures may be too rigid to address the rapid evolution of scientific advancements. This issue is particularly relevant at the academic level: The results from a recent survey showed that researchers face several difficulties in collaborating with regulators, including poor availability and flexibility from ethics committees/regulators for clarification and support (5).

The aim of this study was to analyze, in parallel, the developmental status and the regulatory status of ASs targeting NBL suitable for entering into frontline therapies and for being authorized for pediatric use in the EU, by considering (a) availability of preclinical and clinical studies relevant to a pediatric marketing authorization (MA); (b) availability of an agreed PIP (or of a waiver) and its advancements; and (c) other regulatory procedures applied to the concerned ASs.

Emerging gaps from this analysis and suggestions for improvement were considered and have been discussed also in light of the next revision of the EU Pediatric and Orphan Regulations,^1^ (21).

## Materials and methods

### Study sample

ASs targeting NBL were searched by consulting PubMed (28). The following search terms were adopted: (All field < target therapy > AND All field < neuroblastoma >). Specific filters were applied such as follows: child (birth, 18 years); language (English); article type (review and systematic review); and publication date (January 2018–June 2022). The resulting articles were first screened by the title and abstract to eliminate non-relevant articles (i.e., not focused on NBL or not reporting ASs targeting NBL). The selected articles were processed to derive a list of the ASs for which the following information was available: name of the AS; type of the substance (chemical or biological); and type and description of molecular pathways/genetic aberration targeting NBL. If details were not available, the article was excluded. Immunotherapy and other therapeutic agents were also excluded from the study sample.

### Developmental status

For each AS, the following information was collected from both literature and the available clinical trials database (https://clinicaltrials.gov/) (29): **(**1) preclinical toxicity/safety and other studies that are considered necessary to support pediatric studies and marketing authorization of products (30); (2) clinical trials (CTs) and their phases and aims (pharmacokinetic—PK/pharmacodynamics—PD, studies and dose rationale, efficacy-safety trials); (3) other studies, including extrapolation, modeling, and simulation. When available, information about the status of the study (e.g., recruiting, not recruiting, completed, suspended, terminated, and withdrawn) and the availability of study results was collected. For preclinical studies, in addition to literature, we also consulted the European Public Assessment Reports (EPARs, as available on EMA website) to capture studies already submitted to the regulatory authority during previous MA procedures. Preclinical studies aimed to test the pediatric oncology model and “proof of concept” studies were not considered in this study.

### Regulatory information

For each AS, it was investigated for its inclusion in (1) an MP, approved up to 2022 and their adult and/or pediatric indications; (2) a waiver (i.e., class or product-specific waiver) and the waiver indication; (3) an agreed PIP including indication and advancement status (expected and current time to completion, and compliance check), an age range of the experimental population(s), and also the number and type of studies required (i.e., quality, preclinical, clinical, and other studies); and (4) other regulatory procedures (such as orphan designation and PRIME eligibility, conditional approvals, protocol assistance, or other scientific advices).

All data were collected by consulting the EMA publicly available sources, including EPARs (31), orphan designation list (32), opinions and decisions on PIPs and product-specific waivers (33), a list of products granted eligibility to PRIME (34), and EMA annual reports (35).

Two authors performed the literature search as well as the extraction of information from the selected articles independently. Possible inconsistencies were solved by discussion or by appeal to another researcher.

**Figure 1:**
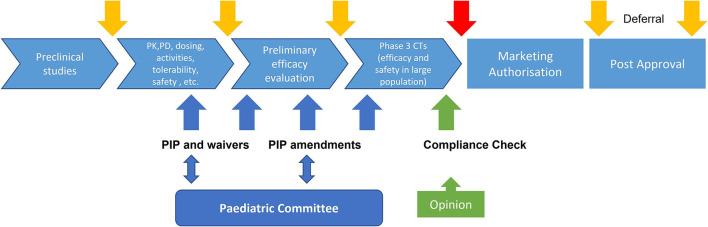
Pediatric drug developmental process.

Figure 1 represents the pediatric drug developmental process for easy understanding.

### Analysis

A descriptive analysis of the results was performed. A comparative analysis was also made among groups of ASs. Indeed, the ASs in the NDDS priority list, based on a high-level scientific process aimed to identify the more promising ASs, are compared with ASs granted with a PIP under the request of a commercial sponsor during a regulatory-based procedure. In addition, the comparison included all the substances that did not receive a PIP and the substances already receiving a MA.

According to the aims of the study, the comparison considered the number of ASs for which the preclinical and clinical studies are available or ongoing, the number and type of regulatory procedures, i.e., PIPs, and the studies' results submitted to the regulatory authorities to be included in the product SmPC.

## Results

### Active substances targeting neuroblastoma: study sample identification and classification

A total of 182 publications resulted from the literature search. Of them, 144 studies were excluded: 32 were not focused on NBL; 44 did not report sufficient details on the molecular NBL target; and 68 were dealing with other therapeutic approaches such as immunotherapy, radiotherapy, and nanomedicine. Therefore, 39 studies (10–17, 20, 36–66) were considered for the analysis. The selection process is shown in Supplementary Figure 1.

**Figure 2:**
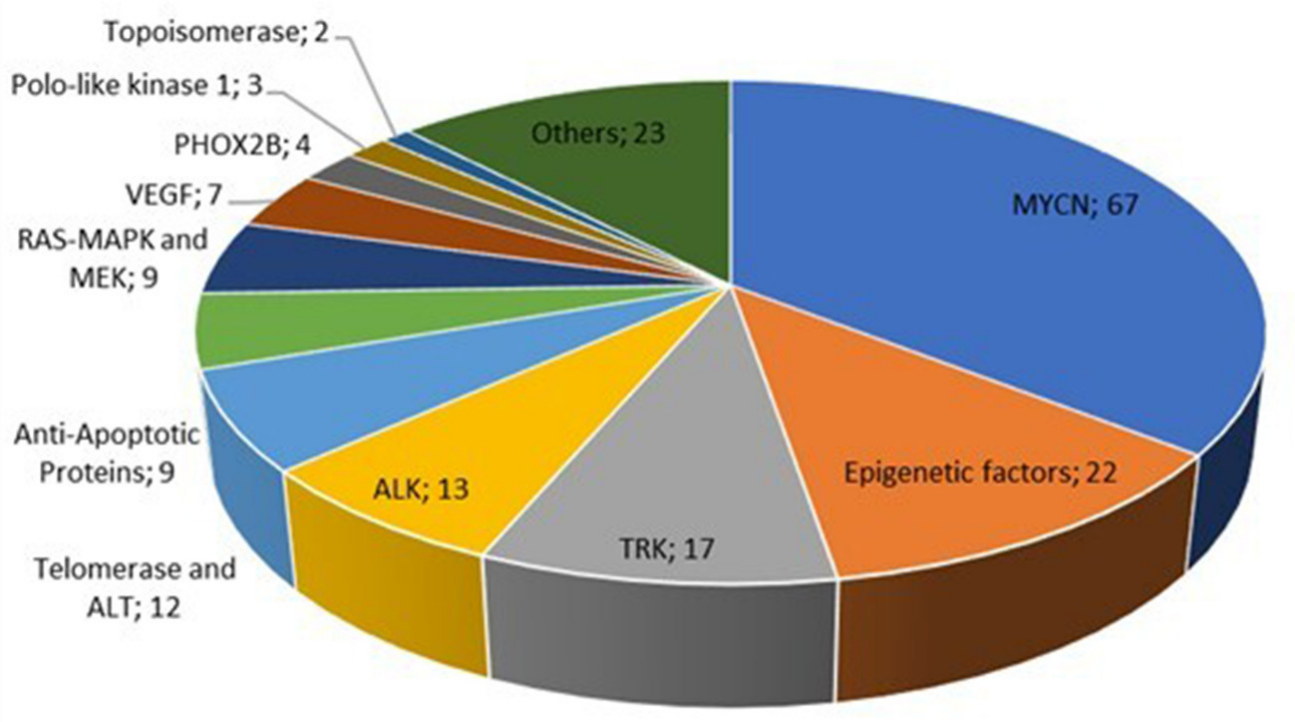
Number of active substances classified by molecular target.

A total of 557 ASs targeting NBL were derived by these studies: 369 were present in more than one study and thus counted only once. The remaining 188 represent the final sample of the study: 185 (98.4%) are chemical agents (small molecules) and three are biologicals. Figure 2 reports the ASs classified by the molecular target. In detail, the ASs targeting N-MYC oncogene are the most represented (67, 35.6%), followed by agents targeting epigenetic factors (22, 11.7%), TRK inhibitors (17, 9%), and the anaplastic lymphoma kinase (ALK) inhibitors (13, 6.9%). The whole list of the identified ASs and their molecular targets is included in the Supplementary material.

### Developmental status

#### Preclinical studies

**Figure 3:**
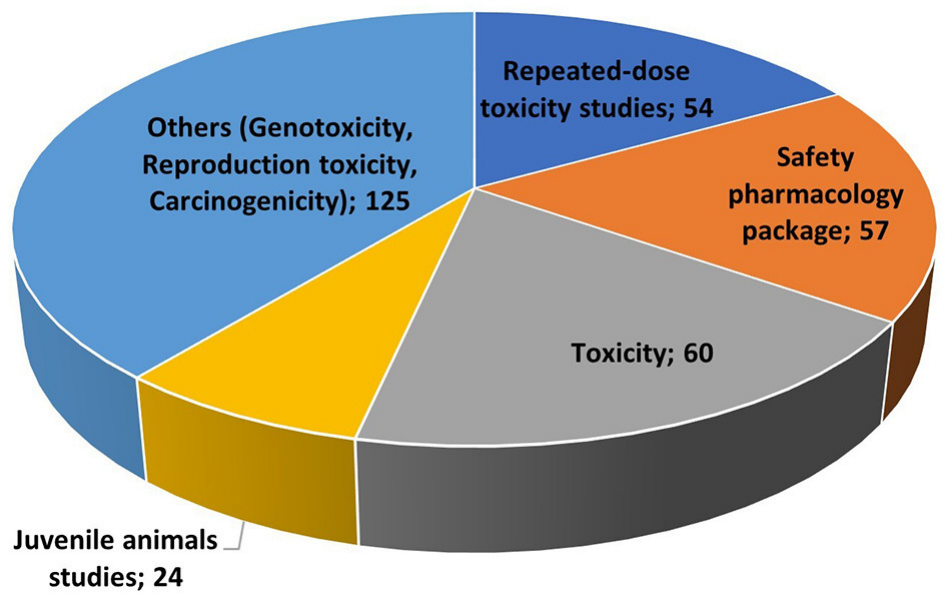
Preclinical studies: type and number.

A total of 319 preclinical studies (i.e., toxicity, safety, and geno/carcinogenicity) were identified involving 115 ASs (Figure 3). Most of the studies were part of the MA dossiers of previously approved MPs. In contrast, 95 studies were not included in a MA and derived from the literature search. As shown in Figure 3, only 24 studies are pediatric-specific (i.e., juvenile animal studies). For 73 ASs, no preclinical studies were found.

#### Clinical studies

**Figure 4:**
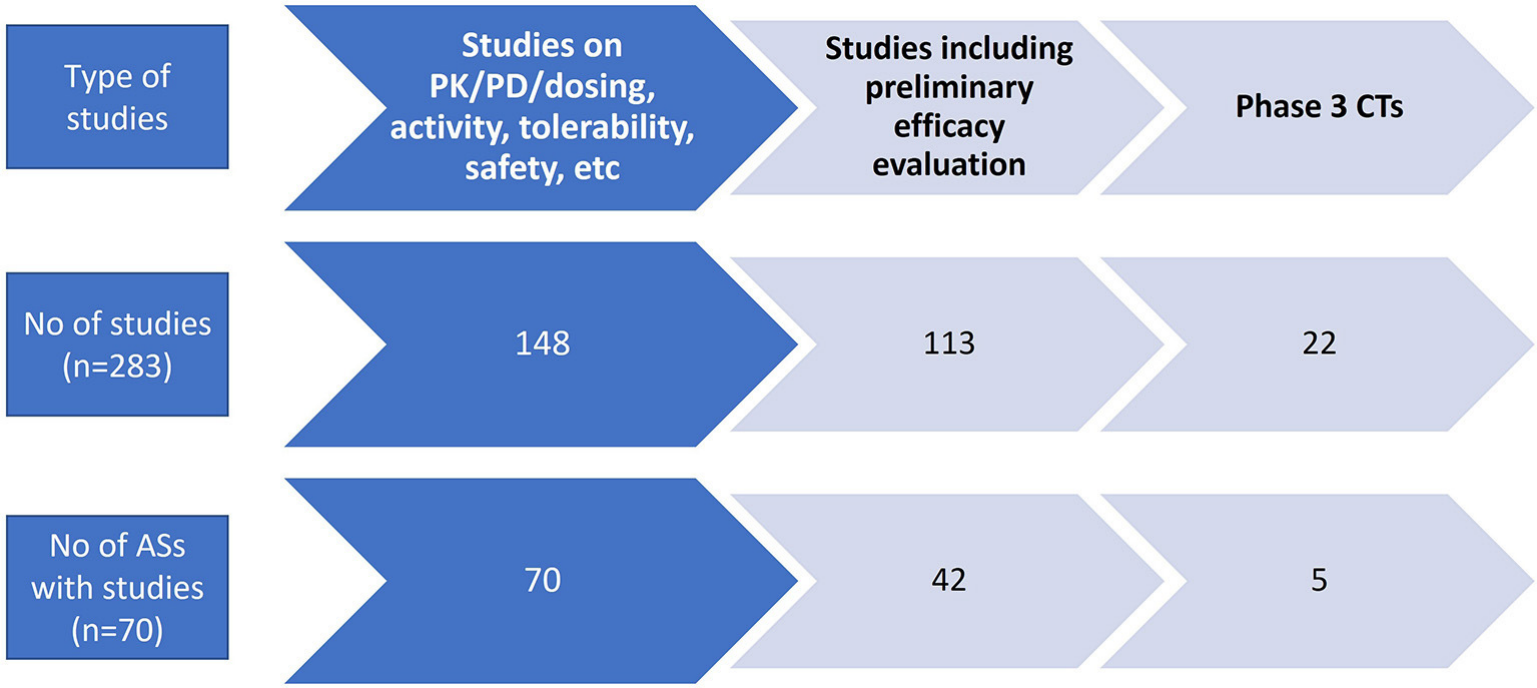
Number of clinical trials and active substances entered in clinical trials.

A total of 283 CTs conducted in NBL patients or in larger oncologic populations having NBL were found for 70 ASs both in monotherapy (60, 21.2%) and in combination (215, 76%). A total of 118 ASs were never included in a CT. In fact, of 118 ASs, 55 were tested neither with preclinical nor with clinical studies. Figure 4 details the type and number of CTs. Most of the CTs are phase 1 up to phase 2 trials (early trials) often covering preliminary efficacy evaluation.

As shown in Figure 4, out of the 283 studies, 52% were phase 1 and 1/2 studies aimed at PK/PD/dosing, activity, tolerability, and safety definition while the remaining CTs also included efficacy as primary or secondary outcomes. Phase 3, including randomized controlled trials (RCTs), were less represented, i.e., 22 RCTs related to 5 ASs, namely, *crizotinib, topotecan, doxorubicin, retinoic acid*, and *etoposide*.

A total of 154 studies were reported as active: recruiting, not yet recruiting, or active not recruiting; 88 (31.1%) were reported as completed; 41 (14.1%) reported as suspended, withdrawn, or no longer available. For 11 (*copanlisib, erlotinib, genistein, HDM201, imetelstat, pazopanib, panobinostat, SF1126, valproic acid, vandetanib*, and *vistusertib*) out of 70 ASs, the development was interrupted.

Considering the type of AS and related target, further considerations were raised. The most represented Ass that entered into the clinical phase belong to the MYCN group (22 of 70, 31.4%). However, in the RAS-MAPK-MEK and VEGF groups, the highest rate of ASs in CTs including efficacy evaluation was observed (i.e., 77.8 and 71.4%, respectively) (Table 1). Supplementary Figure 2 summarizes the developmental status of the ASs in our sample.

**Table 1:** Active substances entered in clinical trials.

**Molecular target**	***N* of ASs**	**ASs with clinical trials**
MYCN	67 (35.6%)	22 (32.8%)
Epigenetic factors	22 (11.7%)	7 (31.8%)
TRK	17 (9%)	8 (47.1%)
ALK	13 (6.9%)	6 (46.1%)
Telomerase and ALT	12 (6.4%)	4 (33.3%)
Anti-apoptotic proteins	9 (4.8%)	2 (22.2%)
RAS-MAPK and MEK	9 (4.8%)	7 (77.8%)
VEGF	7 (3.7%)	5 (71.4%)
PHOX2B	4 (2.1%)	0
Polo-like kinase 1	3 (1.6%)	0
Topoisomerase	2 (1.1%)	2 (100%)
Others	23 (12.2%)	7 (30.4%)
**Total**	**188**	**70**

### Regulatory status

#### Active substances granted with pediatric investigation plans and waivers

A total of 37 ASs (out of 188) were granted a PIP, covering several indications, from the hematolymphoid system (*n* = 13), central nervous system (CNS) tumor (*n* = 6), solid tumor (*n* = 14), and other pediatric malignancies (*n* = 10). The NBL indication was included in 14 PIPs.

A total of 29 PIPs were associated with an approved MP: 28 to an adult MP and 1 to a pediatric-only MP. The remaining eight were PIPs granted to a product under development.

A total of 41 ASs were linked to product-specific waivers or class waivers granted mainly for non-small cell lung cancer, melanoma, and breast cancer indications.

For 18 ASs (43.9%), both PIPs and waivers were granted: the PIPs covered one or more pediatric indications different from the adult ones, and the waivers covered the adult indication; however, seven included NBL.

Noticeably, *trametinib, dabrafenib*, and *binimetinib* have a PIP in the same adult indication (melanoma with BRAF mutation), but other indications targeted by the BRAF mutation of interest for pediatric tumors were also added. Details of ASs granted with both PIPs and waivers for different indications are shown in Table 2.

**Table 2:** Active substances granted with both pediatric investigation plans and waivers.

**ASs**	**Approved adult indication**	**Waived condition**	**Agreed PIP indication**
Abemaciclib	Breast cancer	Breast cancer	1. High-grade glioma, NBL; 2. Ewing's sarcoma
Afatinib	NSCLC	NSCLC	Malignant neoplasms (except hematopoietic and lymphoid tissues neoplasms); CNS malignant neoplasms
Binimetinib	Melanoma with a BRAF V600 mutation	Colorectal cancer	Melanoma with BRAF V600 mutations
Crizotinib	NSCLC	Lung malignant neoplasms	Anaplastic large cell lymphoma and inflammatory myofibroblastic tumors
Olaparib	Ovarian cancer, fallopian tube cancer, and peritoneal cancer	Ovarian cancer, fallopian tube cancer, and peritoneal cancer	Malignant neoplasms (except hematopoietic and lymphoid tissue neoplasms), HRR-mutated solid tumors
Trametinib	Melanoma and NSCLC with BRAF V600 mutation	NSCLC	1. Melanoma with BRAF V600 mutation; 2. Solid tumor with RAS, RAF, or MEK pathway activation; 3. Glioma with BRAF V600 mutation
Venetoclax	CLL and AML	CLL	NBL, acute lymphatic leukemia, acute myeloid leukemia, and non-Hodgkin lymphoma
Carfilzomib	Multiple myeloma	Multiple myeloma	T-cell or B-cell acute lymphoblastic leukemia
Dabrafenib	Melanoma and NSCLC with BRAF V600 mutation	NSCLC	1. Glioma with BRAF V600 mutation; 2. Melanoma with BRAF V600 mutation; 3. Solid tumors with BRAF V600 mutation
Talazoparib	Breast cancer	Breast cancer and prostate malignant neoplasms	Ewing's sarcoma
Imetelstat	Not applicable	Myelofibrosis	AML, myelodysplastic syndromes, and juvenile myelomonocytic leukemia
Erdafitinib	Not applicable	Urothelial carcinoma	Malignant neoplasms, locally advanced or metastatic solid tumors with FGFR alterations, and newly diagnosed solid tumors with FGFR alterations
Palbociclib	Breast neoplasms	Breast malignant neoplasm	Ewing's sarcoma
Ivosidenib	Not applicable	Malignant neoplasms (except CNS tumors, hematolymphoid);	Newly diagnosed or relapsed or refractory AML with an isocitrate dehydrogenase-1 mutation
Veliparib	Not applicable	1. Fallopian tube cancer, ovarian cancer, peritoneal carcinoma; 2. SCLC; 3. breast cancer	Newly diagnosed supratentorial high-grade glioma
Brigatinib	Anaplastic lymphoma kinase (ALK)-positive advanced NSCLC	NSCLC	Newly diagnosed ALK+ anaplastic large cell lymphoma; ALK+ unresectable or recurrent inflammatory myofibroblastic tumors
Ixazomib	Multiple myeloma	Systemic light chain amyloidosis and Multiple myeloma	Lymphoid malignancy (excluding multiple myeloma)
Ruxolitinib	Myelofibrosis, polycythemia vera, and graft-vs.-host disease	Thrombocythemia and polycythemia vera	1. Acute graft-vs.-host disease after allogeneic transplantation 2. Vitiligo 3. Chronic graft-vs.-host disease after allogeneic transplantation

NSCLC, non-small cell lung cancer; SCLC, small cell lung cancer; CLL, chronic lymphocytic leukemia; ALL, acute lymphocytic leukemia; AML, acute myeloid leukemia; NHL, non-Hodgkin lymphoma; CNS, central nervous system.

**Figure 5:**
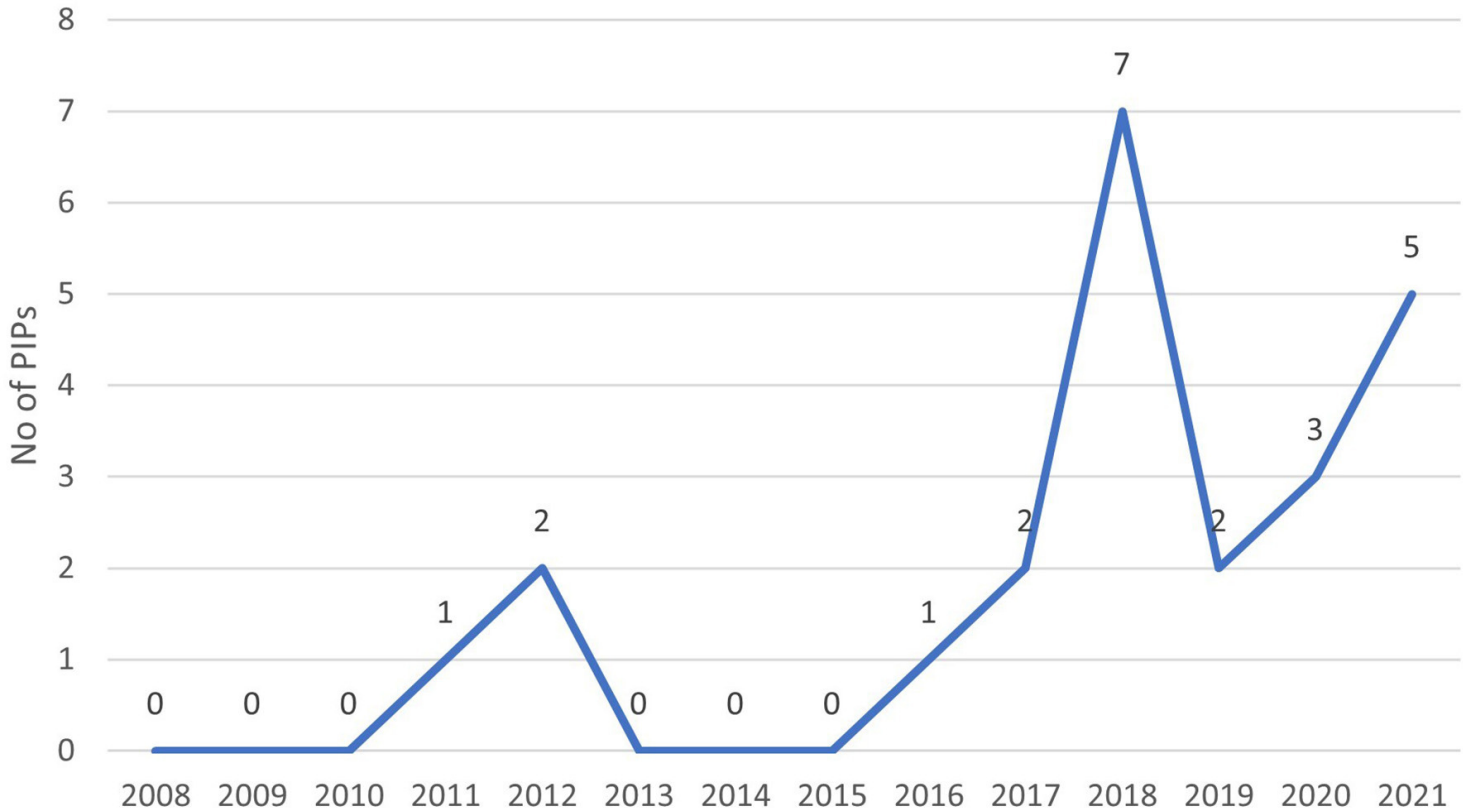
Active substances granted with both pediatric investigation plan and waiver by year.

Most of these PIPs associated with a product covered by a waiver were granted after the EMA revision of the class waivers in 2015 (Figure 5).

#### Pediatric investigation plan contents

The contents of the 14 PIPs that include NBL indication are reported in Supplementary Table 1. In summary, five out of 14 (35.7%) NBL PIPs cover the whole pediatric population from birth, while nine out of 14 (64.3%) cover all ages except younger children (i.e., under 24, 12, 6, or 1 month of age). The PIP indication included NBL and other pediatric solid tumors or, more rarely, hematolymphoid or CNS malignancies.

The total number of (preclinical and clinical) studies required in each PIP varies from two (*afatinib*) to eight (*copanlisib*). In detail, non-clinical studies were required for all PIPs, except for *olaparib*, an ASs already marketed for a different adult indication, and *erdafitinib*. Pediatric-specific studies (i.e., juvenile animal, and age-appropriated formulations) are the most required. The *in vivo/in vitro* pediatric disease model and biomarker studies were required in two PIPs, respectively.

All PIPs included early CTs (i.e., PK and dosing, activity, and safety), and seven of them also included efficacy studies. A phase 3 trial (RCT) was considered for *olaparib*. Extrapolation, modeling, and simulation studies, aimed to support the dosing regimen of the product, were required for six out of 14 PIPs. The expected duration for ongoing PIPs (i.e., the timing between the PIP decision date and the expected PIP completion date, as agreed with PDCO), varies from 2 to 17 years (mean value: 8.5 years). For five out of 14 PIPs (*abemaciclib, copanlisib, idasanutlin, olaparib, and venetoclax*), the expected duration is up to 2027 and, in the case of *olaparib*, it is up to 2035.

The current status of PIPs and their outcomes are described in Table 3: for two ASs (*cobimetinib and afatinib*), the PIP was concluded and received the compliance check after 7.4 and 2.6 years, respectively, while for the other two ASs (*trametinib and dabrafenib*), the studies were completed, but the PIP did not receive a compliance check.

**Table 3:** Pediatric investigation plans—clinical studies, status, and outcomes.

**ASs**	**CC**	**Studies required in the PIPs**	**Studies available (completed or ongoing)**	**Status**	**SmPC variation**
Abemaciclib	No	Dose escalation trial, in combination Study to evaluate safety and efficacy (recruiting)	Dose escalation trial, in combination	Recruiting	
Afatinib	Yes	Dose escalation trial to assess safety, PK, and anti-tumor activity (monotherapy)	Dose escalation trial to assess safety, PK, and anti-tumor activity (monotherapy)	Completed	No changes have been included in the SmPC.
Cobimetinib	Yes	Multiple dose 2-stage trial to evaluate PK, safety, and activity (monotherapy)	Multiple dose 2-stage trial to evaluate PK, safety, and activity (monotherapy)	Completed, negative outcome	No changes have been included in the SmPC.
Copanlisib	No	Dose escalating trial to PK, PD, safety, and activity; safety and efficacy trial (not planned)	Dose escalating trial to PK, PD, safety, and activity	Terminated no anticipated benefits	Product withdrawn
Entrectinib	No	Trial to evaluate PK, safety, and anti-tumor activity monotherapy	Trial to evaluate PK, safety, and anti-tumor activity monotherapy	Active not recruiting	*SmPC Variation:* Treatment of adult and pediatric patients 12 years of age and older
Idasanutlin	No	Trial to evaluate PK, toxicity, safety, and activity; trial to evaluate safety and efficacy	Trial to evaluate PK, toxicity, safety, and activity	Recruiting	
Larotrectinib sulfate	No	Trial to evaluate safety and efficacy still ongoing	Trial to evaluate PK, safety, and anti-cancer activity	Recruiting	*New indication:* Treatment of adult and pediatric patients with solid tumors that display an NTRK gene fusion
Olaparib	No	Study to evaluate safety, tolerability, PK, PD, and preliminary efficacy; multicenter study safety, tolerability, and efficacy (recruiting); randomized, controlled study (not started)	Study to evaluate safety, tolerability, PK, PD, and preliminary efficacy	Recruiting	
Trametinib	No	Dose escalation trial to evaluate safety, tolerability, PK, and PD in combination Bioavailability study in adults	Dose escalation trial to evaluate safety, tolerability, PK, and PD (in combination)	Recruiting	
Venetoclax	No	Dose determination and cohort expansion study; (in combination) Study to evaluate the efficacy	Dose determination and cohort expansion study (in combination)	Active not recruiting	
Dabrafenib	No	Dose escalation trial in combination Bioavailability study in adults	Dose escalation trial (in combination)	Completed	No changes have been included in the SmPC
Erdafitinib	No	Study to evaluate the safety, PK, and anti-tumor activity in pediatric patients Study to assess the safety, PK, and efficacy in pediatric patients and adults	Study to evaluate the safety, PK and anti-tumor activity in pediatric patients Study to assess the safety, PK, and efficacy in pediatric patients and adults	1. Suspended (accrual goal met) 2. Recruiting	
Regorafenib	No	Study to evaluate PK/PD, tolerability, safety, and tumor activity in the pediatric population PK model Study to evaluate the activity, safety, and efficacy	PK/PD, tolerability, safety, and tumor activity (in combination)	Active- not recruiting	
Selpercatinib	No	Studies to evaluate the MTD/recommended dose and dose-limiting toxicities (monotherapy)	Studies to evaluate the MTD/recommended dose and dose-limiting toxicities (monotherapy)	Both recruiting	Preliminary results included in the SmPC

ASs, active substance; CC, compliance check; SmPC, summary of product characteristics; PK, pharmacokinetic; PD, pharmacodynamics; MTD, maximum tolerated dose.

#### Pediatric investigation plan outcomes

We investigated the status of non-clinical and clinical studies foreseen in the PIPs. Several completed preclinical studies, including juvenile animal toxicity studies, resulted in the MA dossiers of the approved MPs. For eight PIPs, clinical studies resulted in active, recruiting, or not recruiting. Development of *copanlisib* was terminated after a phase 1/2 study because of “no anticipated benefit respect to standard therapy”. Negative outcomes were also reported for *cobimetinib* studies.

Modifications in the SmPC were made as follows: for *larotrectinib sulfate and entrectinib*, a new indication, including high-risk NBL, was included in SmPC, based on the preliminary results of the studies and efficacy data extrapolated by other adult studies, respectively; *entrectinib* is authorized for use in children older than 12 years. For *selpercatinib*, the preliminary results were included in the SmPC, without any change of the product indication. It should be noted that, for all these ASs, the related PIPs are still ongoing.

#### Other regulatory procedures

A total of 39 (20.7%) out of 188 ASs were included in the Community Register of designated Orphan Medicinal Products for a total of 59 orphan designations. Only two orphan designations cover the treatment of NBL. *Entrectinib* had an orphan designation, but it was withdrawn in 2018 after the product was designated within the PRIME scheme.

### Developmental and regulatory status comparison

The results of comparison among different groups of ASs are shown in Table 4. The NDDS priority list group has the highest percentage of ASs with CTs and in particular of CTs, including efficacy preliminary data. No results from these NBL studies were included in SmPC. In the group of ASs associated with a PIP, we observed that all the ASs were entered into early CTs, but only a few included efficacy data. When considering the whole group of ASs with a MA, we observed a higher percentage of ASs already submitted to studies, both preclinical and clinical, including efficacy data. In addition, five ASs were granted a pediatric variation in the SmPC: three products, *entrectinib, larotrectinib sulfate*, and *selpercatinib*, are also part of the PIPs group. However, at the time of the SmPC variation, the PIPs were still ongoing. Thus, the variations seem to be independent of the PIP. The other two products *(etoposide* and *doxorubicin)* were approved for a pediatric oncology indication (including NBL) in several member states with national MAs not yet harmonized at the EU level.

**Table 4:** Comparison of the regulatory and developmental status.

	**Preclinical (safety, toxicity)**	**Juvenile animal studies**	**Entered in CTs (PK/PD/dosing, activity, tolerability, safety)**	**Entered in CTs- preliminary efficacy**	**Pediatric variation or results in SmPC**
ASs Associated to a NBL PIPs (*n* = 14)	13 (92.9%)	7 (50%)	14 (100%)	2 (14.3%)	3 (21.4%)
ASs not associated to a PIP (*n* = 151)	81 (53.6%)	9 (6%)	44 (29.1%)	31 (20.5%)	2 (8.7%)
ASs included in NDDS Priority list (*n* = 23)	14 (60.9%)	3 (13%)	22 (95.7%)	12 (52.2%)	0
ASs associated to a MA (*n* = 54)	54 (100%)	21 (38.9%)	40 (74.1%)	25 (46.3%)	5^*^ (9.3%)
ASs not associated to a MA (*n* = 134)	61 (45.5%)	3 (2.2%)	15 (11.2%)	17 (12.7%)	0
ASs in total sample (*n* = 188)	115 (61.2%)	24 (12.8%)	70 (37.2%)	42 (22.3%)	5 (2.7%)

^*^Entrectinib; larotrectinib sulfate; temsirolimus; topotecan; selpercatinib.

ASs, active substances; CTs, clinical trials; PK, pharmacokinetic; PD, pharmacodynamics; SmPC, summary of product characteristics; NBL, neuroblastoma; PIP, pediatric investigation plans; NDDS, neuroblastoma new drug development strategy; MA, marketing authorization.

Finally, there are 44 ASs, not granted a PIP, for which pediatric clinical studies were identified, including also efficacy preliminary data.

## Discussion

There is a larger than expected number of ASs targeting NBL and other pediatric malignancies for which several preclinical and clinical studies have been conducted or are under development. In particular, this number is higher than what is described in the framework of the NDDS forum ended (2020), with 23 genetic targets prioritized out of 40 identified ASs targeting NBL. As a result of our research, 70 ASs entered into the clinical phase; indeed, these studies have been conducted without considering any priority. Owing to the challenges related to the small population and the rarity of the disease (67), it is unlikely that all these ASs, or almost a consistent part of them, will be able to complete a full developmental process up to the inclusion in the frontline or to the market.

Among the ASs included in the clinical phase, ASs targeting/acting on the N-MYC are the most represented and have preclinical and early clinical studies documented in the 46 and 31% of cases, respectively. However, for eight out of 22 ASs of the MYCN group that reached the clinical phase, development was suspended, confirming the several difficulties encountered in moving these drugs to the MA (20). A lower number but a higher percentage of ASs under study were demonstrated for ASs in RAS-MAPK-MEK and VEGF groups. A better outcome may be expected from these studies in the next years.

Our data showed that a large number of ASs (i.e., 115 of 188) have been investigated in preclinical studies, mainly as a part of adult product development, but pediatric-specific studies have been less represented.

On the contrary, it is imperative that the preclinical evaluation is tailored to pediatric specificities and needs, including the characterization of toxicology and safety aspects relevant to the children. Such specificities may have a huge impact on the development of pediatric medicines because it leads regulators to waive the pediatric clinical development for safety reasons, to include contraindications in the SmPC, and to modify/adjust dosing regimen, among others (68). To deal with this issue, an innovative approach is emerging in pediatric drug development, that is exploiting new models, i.e., cells, tissue, organoid, or *in silico* models (69), instead of animal models, under the paradigm that the utility of animal models in place of children for the evaluation of toxicology, efficacy, and safety parameters is very limited or uncertain (70). Moreover, it was suggested that the ability to predict PK and PD with the adoption of new toxicology models (71, 72) or the use of mechanistic simulations may reduce the need and the duration of pediatric clinical studies (73). In particular, preclinical studies on several ASs could help select more specific and efficacious agents on one or more pediatric tumors, facilitating their prioritization.

Improvement in the direction of reducing the number while increasing the efficiency of clinical studies is also expected by the adoption of an innovative clinical study design (more specifically, basket, umbrella, and platform trials) that is more responsive to the complexity of NBL trials (i.e., limited population, genetic and immune phenotypic traits common to other pediatric cancers, and different agents to be studied in combination) (74). Together with a larger application of pharmacometric models and, where possible, extrapolation of existing data, these CT designs may substantially facilitate the progress of more ASs for pediatric use.

As positive results from our regulatory evaluation of ASs targeting NBL, we underline the following:

1. There were more drugs included in a PIP application than in the previous scenario, which was characterized by a higher number of waivers than the agreed PIPs (5).
2. Several ASs for which a waiver was granted for adults indication were also granted with a PIP for a different pediatric oncologic indication, demonstrating that, even with the lack of a specific rule (as the RACE Act in the USA), a medicinal product can be granted both waivers for adult indications and PIP-covering indications of pediatric interest if justified by the MoA of the AS.
3. Through our analysis of the PIPs, we can derive that a combination of preclinical pediatric-specific studies (including formulation and juvenile animal studies) and early clinical studies with limited phase 3 trials is the approach most required by regulators. It may be useful to use this information as a guide for preparing future pediatric developmental plans.
4. Interestingly, out of 14 PIPs with NBL indication, six include extrapolation, modeling, and simulation studies. However, based on the preliminary results of a pilot study on pediatric and rare MPs approved by EMA (75), a significant increase in other computational and innovative statistical models, also including Real Word Data studies, is expected in the next future. This approach will reduce the total economic and resource costs with advantages for patients, avoiding clinical research with limited possibilities to be successful.

However, this analysis also clarifies that several unresolved issues remain, which are as follows:

1. The majority of PIPs and the pediatric variations in SmPC are associated with adult oncology products. This demonstrates that the development of pediatric oncology products is still driven by adult drugs and not by a pediatric-specific interest in the drug.
2. There are still several ASs, even having an MoA of interest for children granted with a waiver for which pediatric development is not required by the EMA. In the EU, it may be necessary to adopt an *ad hoc* rule, such as the RACE Act in the USA, to bridge this gap.
3. The current regulatory procedures seem to be unable to assure faster oncology drug development and its timely approval for the market. As an example, we can consider that *olaparib* received an adult MA in 2014; a PIP for a pediatric indication including NBL was only applied in 2018, with an expected duration for PIP completion of up to 2035. Thus, the pediatric product will (possibly) reach the market 21 years later than the product authorized for adults.
4. Other regulatory procedures applicable to rare and pediatric conditions have been little considered and may be poorly understood by sponsors and researchers (76). As an example, *entrectinib* was withdrawn by the Orphan Designation Registry, while it received the PRIME designation followed by an accelerated approval. This approval was obtained before completing the pediatric development plan and only covers their use in children older than 12 years, leading to high-risk NBL prevailing in younger children.

In conclusion, in pediatric oncology, the number of approved new medicines has not increased significantly during the recent years. With reference to NBL, some old ASs on the market are still used in current practice without including the NBL pediatric indication. Several new ASs have been proposed for development and included in early CTs, but only a few products have been registered for NBL. Considering the several ongoing initiatives,^2^, ^3^,^4^ some improvements may be implemented.

First of all, scientists, regulatory experts, and developers should work together to identify a more efficient, less costly, and time-consuming “pediatric developmental model” integrating predictive preclinical studies and innovative clinical study designs. This model should be proposed and adopted in the PIP application and then agreed by the regulators as fitting with regulatory standards.

Second, the current regulatory process should better support these new scientific paradigms. In particular, some measures proposed in the context of the revision of the European Pediatric and Orphan Regulations may greatly move forward in this direction (77), which are as follows: (a) a drug potentially useful for cancer should be excluded by the application of waiver based on “disease or condition not existing in the pediatric age” and (b) a PIP-staggered approach may be considered. With this approach, the approval of the PIPs will occur step by step and could be stopped or accelerated according to the preliminary results (i.e., predictive preclinical study results) avoiding the extremely long duration of some PIPs; and (c) new incentives specific for products addressing unmet needs for pediatric and rare diseases (i.e., a PRIME-adapted scheme or dedicated research funds) should be largely adopted.

## Data availability statement

The raw data supporting the conclusions of this article will be made available by the authors, without undue reservation.

## Author contributions

AC prepared the first draft of the manuscript, contributed to the design of the research and to set up the methodology, and participated in the analysis of results. RC and AD prepared the first draft of the manuscript and contributed to the design of the research, to perform the research, and to the analysis of the results. AL and LR contributed to the design of the research and provided a general review of the analyzed data. FB and VG provided the final review of the draft. All authors contributed to the article and approved the submitted version.

## Footnotes

^1^Regulation (EC) No 1901/2006 of the European Parliament and of the Council of 12 December 2006 on medicinal products for pediatric use and amending Regulation (EEC) No 1768/92, Directive 2001/20/EC, Directive 2001/83/EC and Regulation (EC) No 726/2004 (Text with EEA relevance). Official Journal of the European Union L 378/.2. 27.12.2006. Available online at: https://eur-lex.europa.eu/legal-content/EN/TXT/PDF/?uri=CELEX:32006R1901&from=EN (accessed November 14, 2022).

^2^SIOP Europe-the European Society for Pediatric Oncology. Available online at: https://siope.eu/ (accessed November 14, 2022).

^3^The Innovative Therapies for Children with Cancer (ITCC) Platform. Available online at: https://www.itccp4.eu/project/overview (accessed November 14, 2022).

^4^European Pediatric Translational Research Infrastructure. Available online at: https://eptri.eu/ (accessed November 14, 2022).

## References

[1] Chung C Boterberg T Lucas J Panoff J Valteau-Couanet D Hero B. Neuroblastoma. Pediatr Blood Cancer. (2021) 68(Suppl. 2):e28473. 10.1002/pbc.28473 33818884 PMC8785544

[2] Brodeur GM Maris JM. Neuroblastoma. In:Pizzo PA Poplack DG, editors. Principles and Practice of Pediatric Oncology. Philadelphia, PA: Lippincott Williams & Wilkins (2015).

[3] Gatta G Botta L Rossi S Aareleid T Bielska-Lasota M Clavel J. Childhood cancer survival in Europe 1999-2007: results of EUROCARE-5—a population-based study. Lancet Oncol. (2014) 15:35–47. 10.1016/S1470-2045(13)70548-5 24314616

[4] Ceci A Felisi M Baiardi P Bonifazi F Catapano M Giaquinto C. Medicines for children licensed by the European Medicines Agency (EMEA): the balance after 10 years. Eur J Clin Pharmacol. (2006) 62:947–52. 10.1007/s00228-006-0193-0 17021892

[5] Toma M Felisi M Bonifazi D Bonifazi F Giannuzzi V Reggiardo G. Paediatric medicines in Europe: the paediatric regulation-is it time for reform? Front Med. (2021) 8:593281. 10.3389/fmed.2021.593281 33604345 PMC7884470

[6] Commission Staff Working Document Evaluation,. Joint Evaluation of Regulation (EC) No 1901/2006 of the European Parliament of the Council of 12 December 2006 on Medicinal Products for Paediatric Use Regulation (EC) No 141/2000 of the European Parliament of the Council of 16 December 1999 on Orphan Medicinal Products. (2020). Available online at: https://eur-lex.europa.eu/legal-content/EN/TXT/?uri=CELEX%3A52020SC0163 (accessed November 14, 2022).

[7] Schoot RA Otth MA Frederix GWJ Leufkens HGM Vassal G. Market access to new anticancer medicines for children and adolescents with cancer in Europe. Eur J Cancer. (2022) 165:146–53. 10.1016/j.ejca.2022.01.034 35235871

[8] Notices from European Union Institutions Bodies Offices Agencies. Council Conclusions on Personalised Medicine for Patients (2015/C 421/03). (2015). Available online at: https://eur-lex.europa.eu/legal-content/EN/TXT/PDF/?uri=CELEX:52015XG1217(01)&from=FR (accessed November 14, 2022).

[9] Nishiwaki S Ando Y. Gap between pediatric and adult approvals of molecular targeted drugs. Sci Rep. (2020) 10:17145. 10.1038/s41598-020-73028-w 33051474 PMC7555892

[10] Zafar A Wang W Liu G Wang X Xian W McKeon F. Molecular targeting therapies for neuroblastoma: progress and challenges. Med Res Rev. (2021) 41:961–1021. 10.1002/med.21750 33155698 PMC7906923

[11] Akter J Kamijo T. How do telomere abnormalities regulate the biology of neuroblastoma? Biomolecules. (2021) 11:1112. 10.3390/biom11081112 34439779 PMC8392161

[12] Greengard EG. Molecularly targeted therapy for neuroblastoma. Children. (2018) 5:142. 10.3390/children5100142 30326621 PMC6210520

[13] Zanotti S Decaesteker B Vanhauwaert S De Wilde B De Vos WH Speleman F. Cellular senescence in neuroblastoma. Br J Cancer. (2022) 126:1529–38. 10.1038/s41416-022-01755-0 35197583 PMC9130206

[14] Li N Spetz MR Li D Ho M. Advances in immunotherapeutic targets for childhood cancers: a focus on glypican-2 and B7-H3. Pharmacol Ther. (2021) 223:107892. 10.1016/j.pharmthera.2021.107892 33992682 PMC8202769

[15] Segura MF Soriano A Roma J Piskareva O Jiménez C Boloix A. Methodological advances in the discovery of novel neuroblastoma therapeutics. Expert Opin Drug Discov. (2022) 17:167–79. 10.1080/17460441.2022.2002297 34807782

[16] Wang J Yao W Li K. Applications and prospects of targeted therapy for neuroblastoma. World J Pediatr Surg. (2020) 3:e000164. 10.1136/wjps-2020-000164 36474924 PMC9716989

[17] Fletcher JI Ziegler DS Trahair TN Marshall GM Haber M Norris MD. Too many targets, not enough patients: rethinking neuroblastoma clinical trials. Nat Rev Cancer. (2018) 18:389–400. 10.1038/s41568-018-0003-x 29632319

[18] Vassal G Rousseau R Blanc P Moreno L Bode G Schwoch S. Creating a unique, multi-stakeholder Paediatric Oncology Platform to improve drug development for children and adolescents with cancer. Eur J Cancer. (2015) 51:218–24. 10.1016/j.ejca.2014.10.029 25434924

[19] Vassal G Houghton PJ Pfister SM Smith MA Caron HN Li XN Shields DJ. International Consensus on minimum preclinical testing requirements for the development of innovative therapies for children and adolescents with cancer. Mol Cancer Ther. (2021) 20:1462–8. 10.1158/1535-7163.MCT-20-0394 34108262

[20] Moreno L Barone G DuBois SG Molenaar J Fischer M Schulte J. Accelerating drug development for neuroblastoma: Summary of the Second Neuroblastoma Drug Development Strategy forum from Innovative Therapies for Children with Cancer and International Society of Paediatric Oncology Europe Neuroblastoma. Eur J Cancer. (2020) 136:52–68. 10.1016/j.ejca.2020.05.010 32653773

[21] FDA. Best Pharmaceuticals for Children Act and Pediatric Research Equity Act Status Report to Congress July 1, 2015 – June 30, 2020. Available online at: https://www.fda.gov/media/157840/download (accessed November 14, 2022).

[22] EMA. Paediatric Investigation Plans. (2010). Available online at: https://www.ema.europa.eu/en/human-regulatory/research-development/paediatric-medicines/paediatric-investigation-plans (accessed November 20, 2022).

[23] FDA. Pediatric Study Plans: Content of and Process for Submitting Initial Pediatric Study Plans and Amended Initial Pediatric Study Plans. (2020). Available online at: https://www.fda.gov/regulatory-information/search-fda-guidance-documents/pediatric-study-plans-content-and-process-submitting-initial-pediatric-study-plans-and-amended (accessed November 20, 2022).

[24] European Commission. Guideline on the format and content of applications for agreement or modification of a paediatric investigation plan and requests for waivers or deferrals and concerning the operation of the compliance check and on criteria for assessing significant studies (Text with EEA relevance). Off J Eur Union. (2014) 279:C 338/1.

[25] EMA. List of Waived Classes of Medicines. (2015). Available online at: https://www.ema.europa.eu/en/human-regulatory/research-development/paediatric-medicines/paediatric-investigation-plans/class-waivers (accessed November 20, 2022)

[26] H.R.2430. FDA Reauthorization Act (FDARA) of 2017. (2017). Available online at: https://www.congress.gov/bill/115th-congress/house-bill/2430/text (accessed November 20, 2022).

[27] European Commission. Regulation (EC) No 141/2000 of the European Parliament and of the council of 16 December 1999 on orphan medicinal products. Off J Eur Commun. (2000) 2201:L18/1.25997721

[28] National Center for Biotechnology Information. National Library of Medicine. Pubmed^®^. (2022). Available online at: https://pubmed.ncbi.nlm.nih.gov/ (accessed November 14, 2022).

[29] National Institutes of Health (NIH). U.S. National Library of Medicines. Clinicaltrials.gov. (2022). Available online at: clinicaltrials.gov. (accessed July 30, 2022).

[30] EMA. EMA/CPMP/ICH/286/1995. ICH Guideline M3(R2) on Non-clinical Safety Studies for the Conduct of Human Clinical Trials and Marketing Authorisation For Pharmaceuticals. (2009). Available online at: https://www.ema.europa.eu/en/documents/scientific-guideline/ich-guideline-m3r2-non-clinical-safety-studies-conduct-human-clinical-trials-marketing-authorisation_en.pdf (accessed November 14, 2022).

[31] EMA. Table of All European Public Assessment Reports (EPAR) for Human and Veterinary Medicines. (2022). Available online at: https://www.ema.europa.eu/sites/default/files/Medicines_output_european_public_assessment_reports.xlsx (accessed October 30, 2022).

[32] EMA. Table of All Orphan Designations. (2022). Available online at: https://www.ema.europa.eu/en/medicines/download-medicine-data#paediatric-investigation-plans-section (accessed October 30, 2022).

[33] EMA. Table of Opinions and Decisions on Paediatric Investigation Plans (PIPs). (2022). Available online at: https://www.ema.europa.eu/en/medicines/download-medicine-data#paediatric-investigation-plans-section (accessed October 30, 2022).

[34] EMA. List of Products Granted Eligibility. (2022). Available online at: https://www.ema.europa.eu/en/human-regulatory/research-development/prime-priority-medicines (accessed October 30, 2022).

[35] EMA. Annual Reports and Work Programmes. European Medicines Agency. (2022). Available online at: https://europa.eu (accessed October 15, 2022).

[36] Aravindan N Herman T Aravindan S. Emerging therapeutic targets for neuroblastoma. Expert Opin Ther Targets. (2020) 24:899–914. 10.1080/14728222.2020.1790528 33021426 PMC7554151

[37] Smith V Foster J. High-risk neuroblastoma treatment review. Children (Basel). (2018) 5:114. 10.3390/children5090114 30154341 PMC6162495

[38] Shimada H Ikegaki N. Genetic and histopathological heterogeneity of neuroblastoma and precision therapeutic approaches for extremely unfavorable histology subgroups. Biomolecules. (2022) 12:79. 10.3390/biom12010079 35053227 PMC8773700

[39] Ciaccio R De Rosa P Aloisi S Viggiano M Cimadom L Zadran SK. Targeting oncogenic transcriptional networks in neuroblastoma: from N-Myc to epigenetic drugs. Int J Mol Sci. (2021) 22:12883. 10.3390/ijms222312883 34884690 PMC8657550

[40] Braoudaki M Hatziagapiou K Zaravinos A Lambrou GI. MYCN in neuroblastoma: “old wine into new wineskins”. Diseases. (2021) 9:78. 10.3390/diseases9040078 34842635 PMC8628738

[41] Kaczmarska A Sliwa P Lejman M Zawitkowska J. The use of inhibitors of tyrosine kinase in paediatric haemato-oncology-when and why? Int J Mol Sci. (2021) 22:12089. 10.3390/ijms222112089 34769519 PMC8584725

[42] Brenner AK Gunnes MW. Therapeutic targeting of the anaplastic lymphoma kinase (ALK) in neuroblastoma-A comprehensive update. Pharmaceutics. (2021) 13:1427. 10.3390/pharmaceutics13091427 34575503 PMC8470592

[43] Pearson ADJ Barry E Mossé YP Ligas F Bird N de Rojas T. Second Paediatric Strategy Forum for anaplastic lymphoma kinase (ALK) inhibition in paediatric malignancies: ACCELERATE in collaboration with the European Medicines Agency with the participation of the Food and Drug Administration. Eur J Cancer. (2021) 157:198–213. 10.1016/j.ejca.2021.08.022 34536944

[44] Mlakar V Morel E Mlakar SJ Ansari M Gumy-Pause F. A review of the biological and clinical implications of RAS-MAPK pathway alterations in neuroblastoma. J Exp Clin Cancer Res. (2021) 40:189. 10.1186/s13046-021-01967-x 34103089 PMC8188681

[45] Ando K Nakagawara A. Acceleration or brakes: which is rational for cell cycle-targeting neuroblastoma therapy? Biomolecules. (2021) 11:750. 10.3390/biom11050750 34069817 PMC8157238

[46] Zafar A Wang W Liu G Xian W McKeon F Zhou J. Targeting the p53-MDM2 pathway for neuroblastoma therapy: Rays of hope. Cancer Lett. (2021) 496:16–29. 10.1016/j.canlet.2020.09.023 33007410 PMC8351219

[47] Jin Z Lu Y Wu Y Che J Dong X. Development of differentiation modulators and targeted agents for treating neuroblastoma. Eur J Med Chem. (2020) 207:112818. 10.1016/j.ejmech.2020.112818 32937281

[48] George SL Parmar V Lorenzi F Marshall LV Jamin Y Poon E. Novel therapeutic strategies targeting telomere maintenance mechanisms in high-risk neuroblastoma. J Exp Clin Cancer Res. (2020) 39:78. 10.1186/s13046-020-01582-2 32375866 PMC7201617

[49] Southgate HED Chen L Curtin NJ Tweddle DA. Targeting the DNA damage response for the treatment of high risk neuroblastoma. Front Oncol. (2020) 10:371. 10.3389/fonc.2020.00371 32309213 PMC7145987

[50] Tucker ER Poon E Chesler L. Targeting MYCN and ALK in resistant and relapsing neuroblastoma. Cancer Drug Resist. (2019) 2:803–12. 10.20517/cdr.2019.009 35582571 PMC8992505

[51] Lima L de Melo TCT Marques D de Araújo JNG Leite ISF Alves CX. Modulation of all-trans retinoic acid-induced MiRNA expression in neoplastic cell lines: a systematic review. BMC Cancer. (2019) 19:866. 10.1186/s12885-019-6081-7 31470825 PMC6717326

[52] Kong X Pan P Sun H Xia H Wang X Li Y. Drug discovery targeting anaplastic lymphoma kinase (ALK). J Med Chem. (2019) 62:10927–54. 10.1021/acs.jmedchem.9b00446 31419130

[53] Pastor ER Mousa SA. Current management of neuroblastoma and future direction. Crit Rev Oncol Hematol. (2019) 138:38–43. 10.1016/j.critrevonc.2019.03.013 31092383

[54] Aubry A Galiacy S Allouche M. Targeting ALK in cancer: therapeutic potential of proapoptotic peptides. Cancers. (2019) 11:275. 10.3390/cancers11030275 30813562 PMC6468335

[55] Umapathy G Mendoza-Garcia P Hallberg B Palmer RH. Targeting anaplastic lymphoma kinase in neuroblastoma. APMIS. (2019) 127:288–302. 10.1111/apm.12940 30803032 PMC6850425

[56] Oh L Hafsi H Hainaut P Ariffin H. p53, stem cell biology and childhood blastomas. Curr Opin Oncol. (2019) 31:84–91. 10.1097/CCO.0000000000000504 30585860

[57] MacFarland S Bagatell R. Advances in neuroblastoma therapy. Curr Opin Pediatr. (2019) 31:14–20. 10.1097/MOP.0000000000000711 30480556

[58] Huang H. Anaplastic lymphoma kinase (ALK) receptor tyrosine kinase: a catalytic receptor with many faces. Int J Mol Sci. (2018) 19:3448. 10.3390/ijms19113448 30400214 PMC6274813

[59] Zage PE. Novel therapies for relapsed and refractory neuroblastoma. Children. (2018) 5:148. 10.3390/children5110148 30384486 PMC6262328

[60] Yuan J Zhang S Zhang Y. Nrf1 is paved as a new strategic avenue to prevent and treat cancer, neurodegenerative and other diseases. Toxicol Appl Pharmacol. (2018) 360:273–83. 10.1016/j.taap.2018.09.037 30267745

[61] Yoshida GJ. Emerging roles of Myc in stem cell biology and novel tumor therapies. J Exp Clin Cancer Res. (2018) 37:173. 10.1186/s13046-018-0835-y 30053872 PMC6062976

[62] Jubierre L Jiménez C Rovira E Soriano A Sábado C Gros L. Targeting of epigenetic regulators in neuroblastoma. Exp Mol Med. (2018) 50:1–12. 10.1038/s12276-018-0077-2 29700278 PMC5938021

[63] Tolbert VP Matthay KK. Neuroblastoma: clinical and biological approach to risk stratification and treatment. Cell Tissue Res. (2018) 372:195–209. 10.1007/s00441-018-2821-2 29572647 PMC5918153

[64] Della Corte CM Viscardi G Di Liello R Fasano M Martinelli E Troiani T. Role and targeting of anaplastic lymphoma kinase in cancer. Mol Cancer. (2018) 17:30. 10.1186/s12943-018-0776-2 29455642 PMC5817803

[65] Janoueix-Lerosey I Lopez-Delisle L Delattre O Rohrer H. The ALK receptor in sympathetic neuron development and neuroblastoma. Cell Tissue Res. (2018) 372:325–37. 10.1007/s00441-017-2784-8 29374774

[66] Valter K Zhivotovsky B Gogvadze V. Cell death-based treatment of neuroblastoma. Cell Death Dis. (2018) 9:113. 10.1038/s41419-017-0060-1 29371588 PMC5833874

[67] Giannuzzi V Conte R Landi A Ottomano SA Bonifazi D Baiardi P. Orphan medicinal products in Europe and United States to cover needs of patients with rare diseases: an increased common effort is to be foreseen. Orphanet J Rare Dis. (2017) 12:64. 10.1186/s13023-017-0617-1 28372595 PMC5376695

[68] EMA. SME workshop: Focus on non-clinical aspects. Pre-clinical Requirements to Support Development of Paediatric Medicines. (2016). Available online at: https://www.ema.europa.eu/en/documents/presentation/presentation-pre-clinical-requirements-support-development-paediatric-medicines-janina-karres_en.pdf (accessed November 20, 2022).

[69] van Berlo D Nguyen VV Gkouzioti V Leineweber K Verhaar MC van Balkom BW. Stem cells, organoids, and organ-on-a-chip models for personalized in vitro drug testing. Curr Opin Toxicol. (2021) 28:7–14. 10.1016/j.cotox.2021.08.006

[70] Bailey GP Mariën D. The value of juvenile animal studies "What have we learned from preclinical juvenile toxicity studies? II” Birth Defects. Res B Dev Reprod Toxicol. (2011) 92:273–91. 10.1002/bdrb.20328 22623019

[71] EMA. EMA/470807/2011 Veterinary Medicines and Product Data Management. Statement of the EMA Position on the Application of the 3Rs (Replacement, Reduction and Refinement) in the Regulatory Testing of Human and Veterinary Medicinal Products. (2011). Available online at: https://www.ema.europa.eu/en/documents/other/statement-european-medicines-agency-position-application-3rs-replacement-reduction-refinement_en.pdf (accessed November 14, 2022).

[72] Gorzalczany SB Rodriguez Basso AG. Strategies to apply 3Rs in preclinical testing. Pharmacol Res Perspect. (2021) 9:e00863. 10.1002/prp2.863 34609088 PMC8491455

[73] Zuang V Dura A Ahs Lopez E Barroso J Batista Leite S Berggren E. Non-animal Methods in Science and Regulation, EUR 30960 EN. Luxembourg: Publications Office of the European Union (2022).

[74] Park JJH Siden E Zoratti MJ Dron L Harari O Singer J Lester RT. Systematic review of basket trials, umbrella trials, and platform trials: a landscape analysis of master protocols. Trials. (2019) 20:572. 10.1186/s13063-019-3664-1 31533793 PMC6751792

[75] Ruggieri L Landi A. Innovative Research Methodologies Application in Paediatric Orphan Medicines XV Foresight Training Course - Boosting Research and Innovation in a Changing Regulatory framework. Bari: Fondazione pr la Ricerca Farmacologica Gianni Benzi Onlus (2022).

[76] Ruggieri L Ceci A Bartoloni F Elie V Felisi M Jacqz-Aigrain E. Paediatric clinical research in Europe: an insight on experts' needs and perspectives. Contemp Clin Trials Commun. (2021) 21:100735. 10.1016/j.conctc.2021.100735 33665471 PMC7905444

[77] European Commission,. Inception Impact Assessment Ref. Ares (2020)7081640 (2020). Available online at: https://eur-lex.europa.eu/legal-content/EN/TXT/?uri=pi_com:Ares(2020)7081640

