# Supplementary material for: Target therapy for high-risk neuroblastoma treatment: integration of regulatory and scientific tools is needed
Source: Front Med (Lausanne). 2023 Jul 14;10:1113460. doi: 10.3389/fmed.2023.1113460 (PMC10377668; doi:10.3389/fmed.2023.1113460)
Supplement: Supplementary file 1 [file Data_Sheet_1.zip › 1.2 Glossary.docx]

Supplementary Material

Glossary

**Active substance:** The substance responsible for the activity of a medicine.

**Approved indication:** A medical condition that a medicine is used for. This can include the treatment, prevention and diagnosis of a disease.

**Centralized Marketing Authorisation procedure:** The European Union-wide procedure for the authorization of medicines, where there is a single application, a single evaluation and a single authorisation throughout the European Union. Only certain medicines are eligible for the centralised procedure.

**Centrally authorized medicinal product:** A medicine with a single marketing authorisation issued by the European Commission and valid across the European Union.

**Clinical trials:** A type of research that studies new tests and treatments and evaluates their effects on human health outcomes.

**Compliance Check:** The verification that some or all studies/measures agreed in a PIP have been conducted in accordance with the PIP decision, including compliance with the agreed timelines for completion of measures.

**Conditional marketing authorization:** The approval of a medicine that addresses unmet medical needs of patients on the basis of less comprehensive data than normally required. The available data must indicate that the medicine’s benefits outweigh its risks and the applicant should be in a position to provide the comprehensive clinical data in the future.

**Drug developmental program (or plan):** The process of bringing a new drug molecule into clinical practice. In its broadest definition this encompasses all steps from the basic research process of finding a suitable molecular target to supporting the commercial launch of the drug.

**Drug Product (US):** The finished dosage form that contains a drug substance, generally, but not necessarily in association with other active or inactive ingredients. It is the equivalent of the EU Medicinal Product.

**Drug substance**: It is the equivalent of the EU Active Substance.

**European public assessment report:** A set of documents describing the evaluation of a medicine authorised via the centralised procedure and including the product information, published on the European Medicines Agency website. European public assessment reports include the product information. Abbreviated as EPAR.

**Marketing Authorization:** The Authorization to put a medicinal product on the market in one, several or all European Union Member States.

**Marketing Authorisation Variation:** A change to the terms of a marketing authorization.

**Mechanism of Action:** A term used to describe how a drug or other substance produces an effect in the body. For example, a drug’s mechanism of action could be how it affects a specific target in a cell, such as an enzyme, or a cell function, such as cell growth. Knowing the mechanism of action of a drug may help provide information about the safety of the drug and how it affects the body. It may also help identify the right dose of a drug and which patients are most likely to respond to treatment. Also called MoA.

**Medicinal Product (EU):** A substance or combination of substances that is intended to treat, prevent or diagnose a disease, or to restore, correct or modify physiological functions by exerting a pharmacological, immunological or metabolic action.

**Orphan designation**: A status assigned to a medicine intended for use against a rare condition. The medicine must fulfil certain criteria for designation as an orphan medicine so that it can benefit from incentives such as protection from competition once on the market.

**Orphan designation withdrawn:** The sponsor of a designated orphan medicine can request removal of its orphan designation from the European Commission's Community register of orphan medicinal products at any time.

**Orphan medicine:** An approved medicinal product intended for the diagnosis, prevention or treatment of a life-threatening or chronically debilitating condition that is rare (affecting not more than five in 10,000 people in the European Union) or where the medicine is unlikely to generate sufficient profit to justify research and development costs.

**Paediatric Committee:** The committee at EMA that is responsible for assessing the content of paediatric investigation plans, which describe how a medicine should be studied in children, as well as waivers. Abbreviated as PDCO.

**Paediatric Investigation Plan:** A development plan aimed at ensuring that the necessary data are obtained through studies in children, to support the authorisation of a medicine for children.

**Pharmacokinetic-Pharmacodynamic (PKPD) analysis**: An alternative to conventional dose-effect analysis, able to relates drug concentration to drug effect.**Phase-I study:** A type of clinical study where a new medicine is given to humans for the first time, usually in healthy volunteers. It looks at the way the medicine is dealt with by the body, its main effects and main side effects.

**Phase-II study:** A type of clinical study conducted after phase I studies to evaluate a medicine’s effects in a particular condition and to determine its common short-term side effects.

**Phase-III study:** A type of clinical study usually conducted in a large group of patients to gather information about a medicine's efficacy and safety, to allow its benefits and risks to be evaluated.

**Personalized medicine:** a medical model using characterisation of individuals’ phenotypes and genotypes (e.g. molecular profiling, medical imaging, lifestyle data) for tailoring the right therapeutic strategy for the right person at the right time, and/or to determine the predisposition to disease and/or to deliver timely and targeted prevention.

**Preclinical study:** The evaluation of potential therapeutic interventions in cells and animals. Drug cndidates for entry into clinical trials can then be selected based on their effectiveness and safety in disease models. All drugs require data from various toxicological preclinical studies to support their potential safety in humans before clinical trials can begin.

**PRIME**: A scheme launched by the European Medicines Agency to enhance support for the development of medicines that target an unmet medical need. This voluntary scheme is based on enhanced interaction and early dialogue with developers of promising medicines, to optimise development plans and speed up evaluation so these medicines can reach patients earlier.

**Protocol assistance:** It is the special form of scientific advice provided from European Medicines Agency, available for developers of designated orphan medicines for rare diseases.

**Scientific advice:** The provision of scientific advice for medicines and medical devices. For medicines, the European Medicines Agency advises on the appropriate tests and studies required in the development or quality of a medicine. For medical devices, the expert panels created by the European Commission advise on clinical studies required in the development of high-risk medical devices.

**Summary of Product Characteristics:** A document, released at the moment of a marketing authorisation, describing the properties and the officially approved conditions of use of a medicine. Summaries of product characteristics form the basis of information for healthcare professionals on how to use the medicine safely and effectively. Abbreviated as SmPC.

**Target Therapy/ies:** Active substances targeting molecular pathways and genetic aberrations in NBL cancer cells.

**Waiver:** An exemption from the obligation to submit a paediatric investigation plan for a new or a in patent medicinal product. Two types of waivers may apply:

1. Class waiver. In this case all medicinal products belonging to a specific therapeutic class such as medicines for diseases that only affect adults, are exempted by the pediatric investigation plan. To this aim a Class waivers list is adopted by the Paediatric Committee (PDCO) and regularly updated.
2. Product specific waiver: An exemption from the obligation to submit paediatric investigation plan, in some or all subsets of the paediatric population for a given condition, route of administration and pharmaceutical form of a specified medicine. Product-specific waivers are adopted by the Paediatric Committee (PDCO).

**Sources:**

# EMA glossary of regulatory terms. <https://www.ema.europa.eu/en/about-us/about-website/glossary>

# FDA glossary of terms. <https://www.fda.gov/patients/clinical-trials-what-patients-need-know/glossary-terms>

# European Commission. Research and Innovation. <https://research-and-innovation.ec.europa.eu/research-area/health/personalised-medicine_en>

# National Institutes of Health. <https://www.nih.gov/health-information/nih-clinical-research-trials-you/glossary-common-terms>

# <http://www.nature.com/subjects/pre-clinical-studies>
