# Supplementary material for: Target therapy for high-risk neuroblastoma treatment: integration of regulatory and scientific tools is needed
Source: Front Med (Lausanne). 2023 Jul 14;10:1113460. doi: 10.3389/fmed.2023.1113460 (PMC10377668; doi:10.3389/fmed.2023.1113460)
Supplement: Supplementary file 1 [file Data_Sheet_1.zip › 2.2 Supplementary Tables.docx]

Supplementary Material

## Supplementary Tables

**Supplementary Table 1.** Pediatric Investigation Plans contents, timing and outcomes.

| **ASs** | **Age groups** | **PIP indications** | **Studies required** | **Expected Completion Date** | **Compliance Check** |
| --- | --- | --- | --- | --- | --- |
| Abemaciclib | birth-18 years | High-grade glioma, NBL | 2 quality | June 2028 | No |
|  |  |  | 1 preclinical (Juvenile toxicity) |  |  |
|  |  |  | 2 clinical |  |  |
|  |  |  | 1 Modeling & Simulation |  |  |
| Afatinib | 1 years -18 years | Malignant neoplasms (except hemato lymphoid neoplasms); Central Nervous System malignant neoplasms | 1 preclinical (Biomarker) | December 2020 | Yes |
|  |  |  | 1 clinical |  |  |
| Cobimetinib | 6 months-18 years | Malignant neoplasms (except haemato lymphoid tissue) with known or expected Ras, Raf or MEK pathway activation | 1 quality | July 2020 | Yes |
|  |  |  | 2 preclinical (Juvenile toxicity and model testing |  |  |
|  |  |  | 1 clinical |  |  |
| Copanlisib | 6 months-18 years | Malignant neoplasms (except haemato lymphoid tissue) relapsed or refractory NBL;Ewing sarcoma, osteosarcoma or rhabdomyosarcoma | 4 preclinical (Pharmacology testing  and tolerability) | December 2027 | No |
|  |  |  | 2 clinical |  |  |
|  |  |  | 2 Modeling & Simulation |  |  |
| Entrectinib | birth-18 years | Malignant neoplasms (except haemato and lymphoid tissue); NTRK fusion-positive locally advanced or metastatic | 2 quality | December 2022 | No |
|  |  |  | 2 preclinical (Juvenile toxicity) |  |  |
|  |  |  | 1 clinical |  |  |
|  |  |  | 1 Modelling & Simulation |  |  |
| Idasanutlin | birth-18 years | Malignant neoplasms (except haemato and lymphoid tissue); Solid malignant tumour newly diagnosed and metastatic, or refractory | 1 quality | May 2029 | No |
|  |  |  | 1 preclinical (Juvenile toxicity) |  |  |
|  |  |  | 2 clinical |  |  |
|  |  |  | 1 Modeling & Simulation |  |  |
| Larotrectinib Sulfate | birth-18 years | Malignant neoplasms (except Central Nervous System tumours, haemato lymphoid neoplasms); Solid tumours harboring an NTRK fusion | 2 quality | September 2021 | No |
|  |  |  | 2 preclinical (Juvenile toxicity) |  |  |
|  |  |  | 1 clinical |  |  |
|  |  |  | 1 Modelling & Simulation |  |  |
| Olaparib | 6 months-18 years | Malignant neoplasms (except hematopoietic and lymphoid tissue neoplasms), HRR mutated solid tumors | 1 quality | December 2035 | No |
|  |  |  | 3 clinical |  |  |
| Trametinib | 1 months-18 years | Melanoma with BRAF V600 mutation; Solid malignant tumors with RAS, RAF or MEK pathway activation; | 1 quality | June 2022 | No |
|  |  |  | 1 preclinical (Juvenile toxicity) |  |  |
|  |  |  | 2 clinical |  |  |
| Venetoclax | birth-18 years | NBL, hematopoietic and lymphoid tissue neoplasm | 1 quality | December 2029 | No |
|  |  |  | 1 preclinical (Juvenile toxicity) |  |  |
|  |  |  | 2 clinical |  |  |
| Dabrafenib | 1 years -18 years | Melanoma with BRAF V600 mutation; Solid malignant tumors with BRAF V600 mutation | 1 quality | June 2022 | No |
|  |  |  | 3 preclinical (Juvenile toxicity) |  |  |
|  |  |  | 2 clinical |  |  |
| Erdafitinib | 2 years-18 years | Malignant solid neoplasms; Locally advanced or metastatic and newly diagnosed solid tumors with FGFR alterations | 1 quality | June 2029 | No |
|  |  |  | 2 clinical |  |  |
| Regorafenib | 6 months-18 years | Malignant neoplasms (except hematopoietic and lymphoid tissue) | 1 quality | December 2024 | No |
|  |  |  | 2 preclinical (1 Juvenile toxicity) |  |  |
|  |  |  | 3 clinical |  |  |
| Selpercatinib | 6 months-18 years | RET-mutant medullary thyroid cancer; RET-altered, locally advanced or metastatic, solid tumors or primary Central Nervous System tumors | 1 quality | June 2023 | No |
|  |  |  | 4 preclinical (Juvenile toxicity) |  |  |
|  |  |  | 2 clinical |  |  |

# 
