# Supplementary material for: Target therapy for high-risk neuroblastoma treatment: integration of regulatory and scientific tools is needed
Source: Front Med (Lausanne). 2023 Jul 14;10:1113460. doi: 10.3389/fmed.2023.1113460 (PMC10377668; doi:10.3389/fmed.2023.1113460)
Supplement: Supplementary file 1 [file Data_Sheet_1.zip › Supplementary_Material_list.docx]

Supplementary Material

**Target therapy for high-risk neuroblastoma treatment: integration of regulatory and scientific tools is needed**

**Adriana Ceci*, Rosa Conte†, Antonella Didio†, Annalisa Landi, Lucia Ruggieri, Viviana Giannuzzi and Fedele Bonifazi**

*** Correspondence:** Adriana Ceci: [adriceci.uni@gmail.com](mailto:adriceci.uni@gmail.com)

†These authors have contributed equally to this work and share first authorship

# Supplementary Data

## List of the selected active substances targeting neuroblastoma

## Glossary

# Supplementary Figures and Tables

## Supplementary Figures

**Supplementary Figure 1.** Flowchart: literature search results.

**Supplementary Figure 2.** ASs developmental status.

## Supplementary Tables

**Supplementary Table 1.** Pediatric Investigation Plans contents, timing and outcomes.
