# Supplementary figures and images for: Target therapy for high-risk neuroblastoma treatment: integration of regulatory and scientific tools is needed
Source: Front Med (Lausanne). 2023 Jul 14;10:1113460. doi: 10.3389/fmed.2023.1113460 (PMC10377668; doi:10.3389/fmed.2023.1113460)

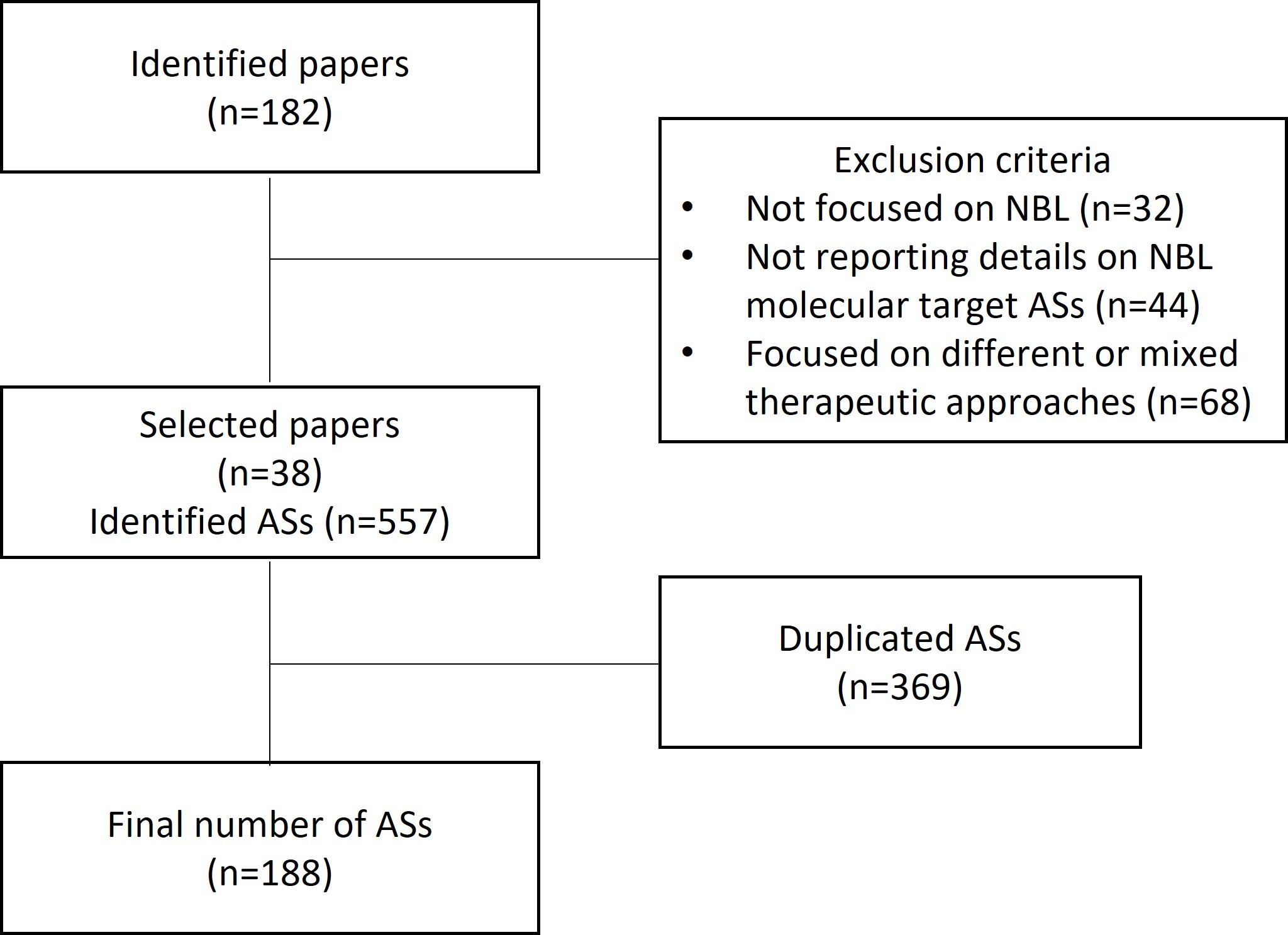

Supplement: Supplementary file 1 [file Data_Sheet_1.zip › Supplementary Figure 1.jpg]

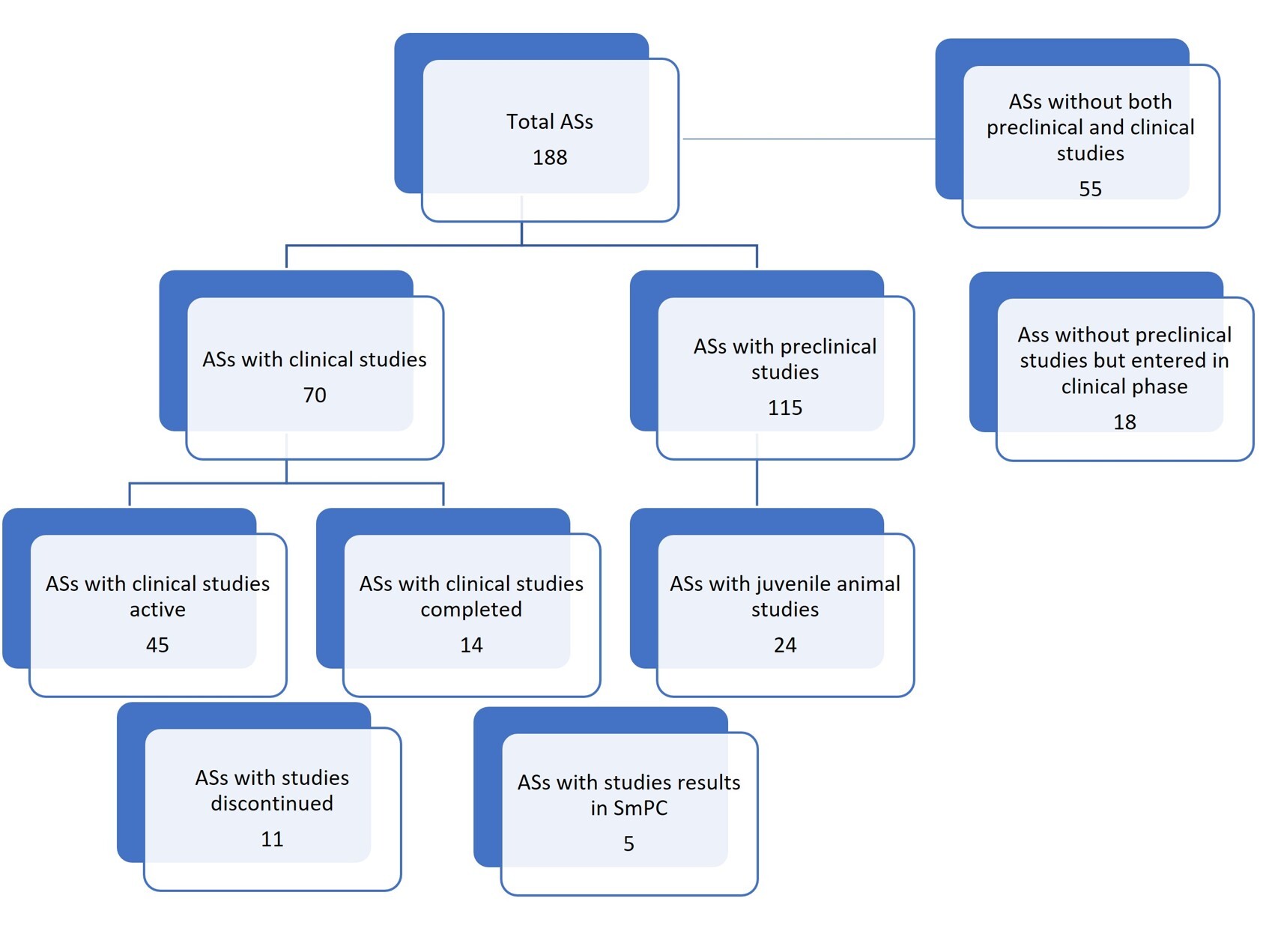

Supplement: Supplementary file 1 [file Data_Sheet_1.zip › Supplementary Figure 2.jpg]
